# *In situ* crystal data-collection and ligand-screening system at SPring-8

**DOI:** 10.1107/S2053230X22005283

**Published:** 2022-05-27

**Authors:** Hideo Okumura, Naoki Sakai, Hironori Murakami, Nobuhiro Mizuno, Yuki Nakamura, Go Ueno, Takuya Masunaga, Takashi Kawamura, Seiki Baba, Kazuya Hasegawa, Masaki Yamamoto, Takashi Kumasaka

**Affiliations:** a Structural Biology Division, Japan Synchrotron Radiation Research Institute, 1-1-1 Kouto, Sayo-cho, Sayo-gun, Hyogo 679-5198, Japan; b Life Science Research Infrastructure Group, RIKEN SPring-8 Center, 1-1-1 Kouto, Sayo-cho, Sayo-gun, Hyogo 679-5148, Japan

**Keywords:** protein crystallography, *in situ* X-ray diffraction, room-temperature data collection, crystallization plates, ligand screening

## Abstract

*In situ* diffraction data collection using crystallization plates has been utilized for macromolecules to evaluate crystal quality without requiring additional sample treatment such as cryocooling. Although it is difficult to collect complete data sets using this technique due to the mechanical limitation of crystal rotation, recent advances in methods for data collection from multiple crystals have overcome this issue. At SPring-8, an *in situ* diffraction measurement system was constructed consisting of a goniometer for a plate, an articulated robot and plate storage. Using this system, complete data sets were obtained utilizing the small-wedge measurement method. Combining this system with an acoustic liquid handler to prepare protein–ligand complex crystals by applying fragment compounds to trypsin crystals for *in situ* soaking, binding was confirmed for seven out of eight compounds. These results show that the system functioned properly to collect complete data for structural analysis and to expand the capability for ligand screening in combination with a liquid dispenser.

## Abstract 2

An *in situ* X-ray diffraction measurement system using a crystallization plate has been constructed at the SPring-8 protein crystallography beamline. Utilizing small-wedge measurements and incorporating a liquid dispenser to prepare protein–ligand complex crystals, this system will make ligand screening possible.

## 1. Introduction

In recent years, the efficiency of protein crystallography has improved rapidly. In particular, automated measurement of cryogenic samples has been achieved by using microbeams, sample-exchange robots and high-speed pixel-array detectors in synchrotron-radiation facilities, which has greatly contributed to the expansion of the number of successful structural analyses (Owen *et al.*, 2016 ▸). The combination of the automation of diffraction data collection and the automation of data processing has made it possible to acquire a large amount of data in a short period of time. Automated systems have been developed at the SPring-8 protein crystallography beamlines (Ueno *et al.*, 2005 ▸; Yamashita *et al.*, 2018 ▸; Hirata *et al.*, 2019 ▸; Murakami *et al.*, 2020 ▸; Nakamura *et al.*, 2020 ▸). Cryogenic measurements have become the standard method for high-throughput measurement, even though handling a large number of crystals for crystal cryocooling remains a major issue in automation (Wright *et al.*, 2021 ▸).

Furthermore, synchrotron microbeam technology enables small-wedge data collection from multiple tiny crystals or multiple domains within each crystal (Cherezov *et al.*, 2007 ▸; Rasmussen *et al.*, 2011 ▸; Rosenbaum *et al.*, 2011 ▸). This technique has been further developed into serial synchrotron rotation crystallography (SS-ROX; Gati *et al.*, 2014 ▸; Hasegawa *et al.*, 2017 ▸, 2021 ▸). The small-wedge data-collection method is useful in the case of multiple tiny crystals, which limit the X-ray dose due to the small volume of each crystal. The approach is important for room-temperature data collection because the dose limitation at room temperature is lower than that at cryo temperature.

In the situation described above, the *in situ* diffraction data-collection technique using crystallization plates, also known as the crystallization plate scan method, has been developed (Aller *et al.*, 2015 ▸; Martiel *et al.*, 2018 ▸) to evaluate the quality of the crystals obtained. Incorporating crystallization plates using this method provides the advantages of not requiring soaking in cryoprotectant agents or picking up the crystals in a cryo-loop. Various limitations imposed by the crystallization plate include narrower oscillation angles in the diffraction system, a higher background due to the presence of scatterers such as crystallization plates, seals and solvents, and lower dose limits at room temperature. However, the small-wedge or SS-ROX method can be applied to *in situ* diffraction data collection using crystallization plates; as a result, it is possible to obtain data sets within the constraints of the plate, such as the rotation range.

While much effort has been made to automate the fishing of crystals from crystallization drops for cryoprotection, it is still a labor-intensive process. Since the *in situ* data-collection system can eliminate the crystal-fishing process, it has become suitable for the analysis of hundreds to thousands of ligand–target protein complexes, such as for the screening of compounds that bind to drug-target proteins (Blundell & Patel, 2004 ▸). The demand for efficient protein crystallography is increasing due to the spread of so-called fragment-based drug discovery (Erlanson *et al.*, 2016 ▸), which aims to obtain multiple compounds and design novel molecular scaffolds based on structural information on target proteins in complex with a compound. The screening pipelines for introducing many types of compounds into crystals, such as XChem at Diamond Light Source (Douangamath *et al.*, 2021 ▸) and FragMAX (Lima *et al.*, 2020 ▸) at MAX-IV, are attached to the synchrotron-radiation beamline. At Diamond Light Source, a large number of compounds are efficiently introduced into crystals by using an acoustic liquid handler to inject a small amount of a compound solution directly into the crystallization drops.

At SPring-8, we introduced an acoustic liquid handler in the development of a compound-screening pipeline combined with an automated data-collection system. In this pipeline, the introduction of compounds into crystals is performed by an acoustic liquid handler, enabling a variety of compounds to be introduced directly into crystals in crystallization drops on a 96-well crystallization plate within minutes.

In this paper, we report the development of an *in situ* diffraction measurement system using crystallization plates at SPring-8, which is combined with compound dispensing using an acoustic liquid handler. We assessed its application to compound-screening applications by collecting small-wedge diffraction data. Using this system, we attempted to collect small-wedge data from crystals in multiple drops of various compounds and performed structural analysis. In the experiment, we obtained small-wedge diffraction data from crystals of trypsin in complex with the compounds introduced by the acoustic liquid handler. As a result, we succeeded in structural analysis of complexes with seven of eight tested compounds.

## 2. Equipment and usage

**Figure 1:**
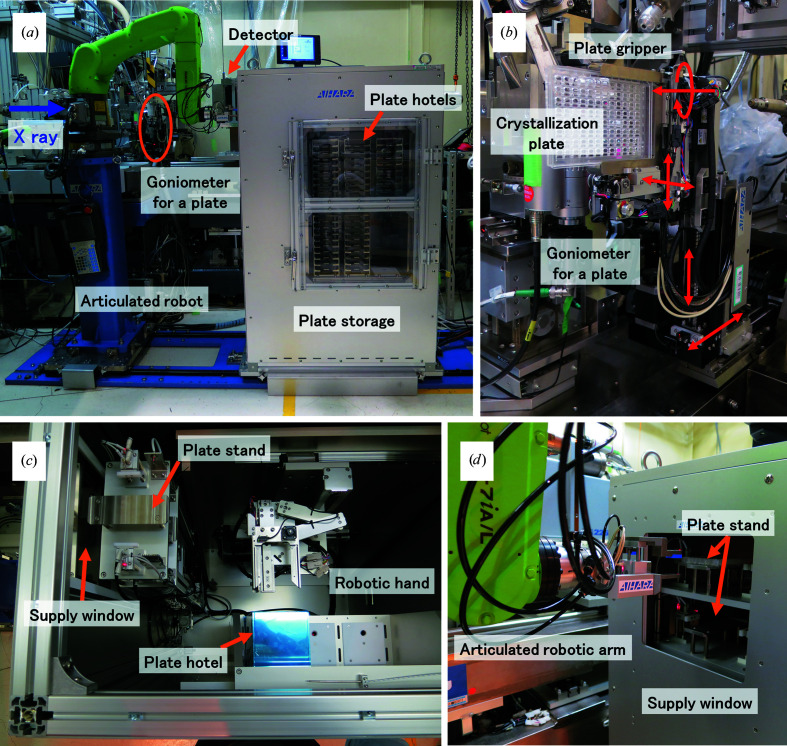
*In situ* diffraction measurement system using a crystallization plate installed in the experimental hutch at BL26B1 at SPring-8. (*a*) The articulated robot for crystallization plate transfer and plate storage. The circle indicates the position of the goniometer for a plate. (*b*) The goniometer for a plate. Red arrows indicate the rotation axis and the directions of movement of each axis. (*c*) Crystallization plates are transferred from a plate hotel to a plate stand by the robotic hand inside the plate storage. (*d*) Supply window on the side of the plate storage.

The *in situ* diffraction measurement system using crystallization plates installed in the BL26B1 experimental hutch (Fig. 1 ▸ *a*) consists of a goniometer with a gripper which holds a crystallization plate (assembled by Rigaku Aihara Seiki Co. Ltd), plate storage (Rigaku Aihara Seiki Co. Ltd) and an articulated robot which has six axes (CR-7iA, FANUC Corporation) for transporting crystallization plates. During the initial development of the SPring-8 *in situ* diffraction measurement system using crystallization plates, the aim of the design was to determine whether the crystals grown in crystallization screening conditions were salt or protein and to confirm the quality of the crystals if they were protein. To efficiently check a large number of crystallization plates, the mounting and dismounting of crystallization plates, as well as the opening and closing of the experimental hutch door each time a crystallization plate is changed, were considered to be bottlenecks to the measurement speed. Therefore, to perform crystallization-plate exchange automatically by submitting commands, we constructed an automatic plate-transport system by installing plate storage and an articulated robot to transport crystallization plates between the plate storage and the goniometer for a plate.

The goniometer for a plate is designed to hold an ANSI/SBS standard-size crystallization plate for both normal height plates of approximately 15 mm and low-profile plates, with a pneumatic plate gripper that opens and closes vertically (Fig. 1 ▸ *b*). In practice, commercially available crystallization plates designed for *in situ* diffraction data collection, such as the In Situ-1 Crystallization Plate (MiTeGen), CrystalQuick X plate (Greiner Bio-One) and CrystalDirect plate (MiTeGen), are assumed to be used. The goniometer for a plate is equipped with an X-ray direction axis (movable range 20 mm), a horizontal axis (movable range 150 mm), a vertical axis (movable range 80 mm) and another vertical axis that is used to determine the height of the rotation axis and evacuate the goniometer for a plate. This goniometer is also equipped with a horizontal rotation axis for oscillation measurements, which has an effective range of −5° to 9°. This limitation of effective range comes from interference with peripheral devices (various parameters are shown in Table 1 ▸).

The plate storage is in charge of storing crystallization plates for diffraction measurements and supplying crystallization plates to the articulated robot for transportation (Fig. 1 ▸ *a*). We used a Rock Imager 54 protein crystallization imager (Formulatrix) as the observation system for crystallization plates in the wet laboratory. This equipment can store six plate hotels (Formulatrix) that each hold nine plates, totaling 54 plates. Our plate-storage capacity was also designed to store six plate hotels, so that it can store all of the crystallization plates within plate hotels stored in the Rock Imager 54. The storage is equipped with a robotic hand that carries the crystallization plate in the storage system using its two automated linear axes and one automated rotational axis (Fig. 1 ▸ *c*). When a crystallization plate is transported from the plate storage to the goniometer for a plate, the robotic hand in the storage takes the crystallization plate out from the back side of the plate hotel, *i.e.* the opposite side of the plate hotel from where the operator places the crystallization plate, and carries it to the plate stand in the plate storage. The supply window on the side of the plate storage opens automatically to enable the crystallization plate to be supplied, and the articulated robot picks the crystallization plate up from the plate stand of the storage and then transports the crystallization plate to the goniometer for a plate (Fig. 1 ▸ *d*). The temperature inside of the storage is controlled at 20°C by air introduced from a precision air conditioner (PAU-300S, Apiste Corporation, Japan).

The articulated robot for crystallization plate transfer and the plate storage are installed in the experimental hutch of BL26B1, and the robot automatically exchanges the crystallization plates during the X-ray diffraction experiment. We adopted a small collaborative robot that is programmed to work safely and to stop when it comes into physical contact with people or peripheral devices such as a detector. The operation of devices whose motion trajectories may overlap with the robot, such as the sample changer and the detector, are restricted by interlocks during robot operation to reduce the risk of collisions. The articulated robot has six axes and has the function of receiving the crystallization plate supplied from the plate storage and transporting it to the goniometer for a plate promptly. The robotic hand attached to the tip of the articulated robot arm was newly designed for holding the crystallization plate, and it is driven by air pressure. A supplementary movie showing the robot in action is available in the supporting information.

**Figure 2:**
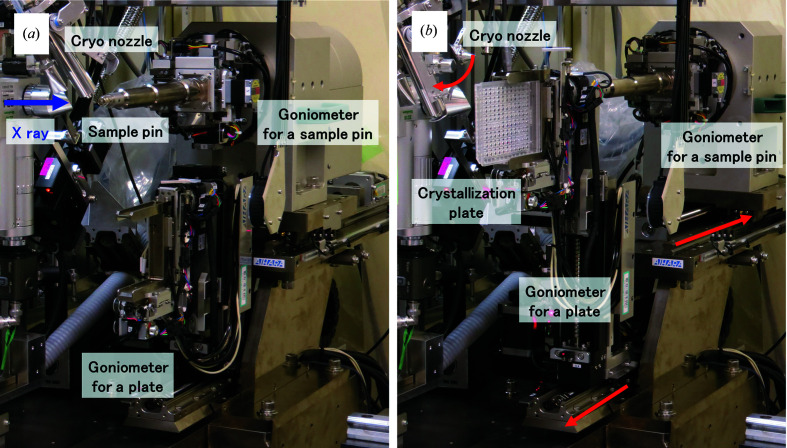
Two switchable measurement modes. (*a*) Diffraction measurement mode using the goniometer for a sample pin for cryogenic measurement. (*b*) Crystallization plate diffraction measurement mode. Red arrows indicate the direction of movement of the goniometer for a sample pin, the goniometer for a plate and the cryo nozzle.

During diffraction measurements using a sample pin in the cryogenic experiment, the goniometer for a plate is tucked away diagonally below the goniometer for a sample pin. For *in situ* diffraction measurements, the goniometer for a sample pin is moved backwards by an automated axis under its body, the cryo nozzle is evacuated to a position where it does not interfere with a crystallization plate and the goniometer for a plate is then moved to the X-ray exposure position (Fig. 2 ▸).

**Figure 3:**
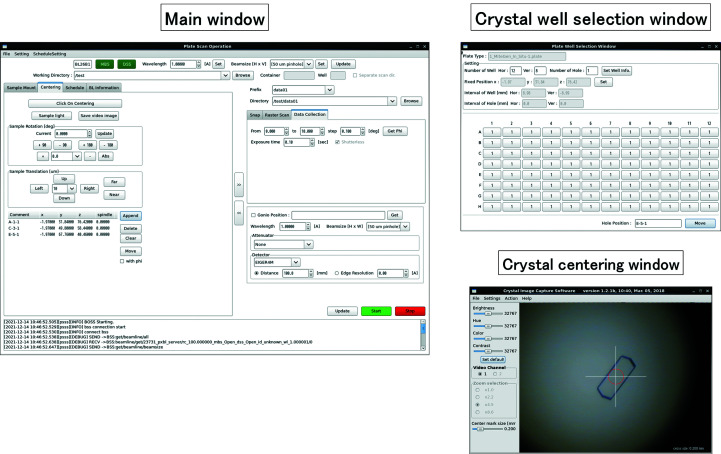
The graphical user interface dedicated to the plate diffraction measurements. It consists of a main operation window, a crystal well selection window and a crystal centering window.

When performing *in situ* diffraction measurements using a crystallization plate, the articulated robot and the plate storage are controlled by the dedicated device server, and the measurement instruments are controlled by the *Beamline Scheduling Software* (*BSS*; Ueno *et al.*, 2005 ▸). The plate scan operation software GUI submits commands to the dedicated device server and *BSS*. Fig. 3 ▸ shows a snapshot of the plate scan operation software GUI, which consists of a main window, a crystal well selection window and a crystal centering window. The main window allows the operators to exchange the crystallization plate, to select the plate type from the list which is registered, to set the measurement conditions and to perform the measurement. Measurements can be started immediately after centering a crystal, or multiple crystal positions can be registered for later sequential measurements. The crystal well selection window is provided so that operators can easily move to the target well by selecting the well number on the crystallization plate at any time except during plate transfers or diffraction measurements. In conjunction with the crystal centering window, the sample crystals can be observed by a coaxial microscope. Centering of the crystals is performed by aligning the horizontal axis and the vertical axis by double-clicking the crystal image. Centering along the beam direction is decided with the focus of the crystal image. The positional deviation due to rotation is adjusted in advance to be less than a few micrometres at 14° rotation.

## 3. Evaluation of the measurement system by diffraction experiments

To evaluate this *in situ* diffraction measurement system using a crystallization plate, we performed small-wedge measurements at room temperature using three types of protein crystals, lysozyme, thermolysin and trypsin, and analyzed the data obtained from each. Lysozyme (egg-white lysozyme) crystals were prepared using the following procedure. Powdered lysozyme (catalog No. L6876, Sigma–Aldrich) was dissolved to 20 mg ml^−1^ in 10 m*M* acetic acid pH 4.6 buffer solution. The crystallization conditions were 100 m*M* citrate–NaOH pH 4.4, 1.0 *M* NaCl. Crystallization drops of 200 nl protein solution mixed with 200 nl crystallization solution were used for crystallization. The sizes of the lysozyme crystals used for measurement were ∼80–200 µm. To crystallize thermolysin, powdered thermolysin (catalog No. 201-08331, FUJIFILM Wako Pure Chemical Corporation, Japan) was dissolved to 25 mg ml^−1^ in 10 m*M* sodium hydroxide. The crystallization conditions were 100 m*M* MES–NaOH pH 6.5, 15%(*w*/*v*) PEG 2000 MME, 5 m*M* CaCl_2_ (Birrane *et al.*, 2014 ▸). Crystallization drops of 200 nl protein solution mixed with 200 nl crystallization solution were used for crystallization. The sizes of the thermolysin crystals used for measurement were ∼100–300 µm. For the crystallization of trypsin (bovine pancreatic trypsin), powdered trypsin was dissolved to 30 mg ml^−1^ in 25 m*M* HEPES pH 7.0, 5 m*M* CaCl_2_ buffer. The crystallization conditions were 100 m*M* Tris–HCl pH 8.5, 30%(*w*/*v*) PEG 3350, 200 m*M* Li_2_SO_4_ (Yamane *et al.*, 2011 ▸). Crystallization drops of 250 nl protein solution mixed with 250 nl crystallization solution were used. The sizes of the trypsin crystals used for measurement were ∼80–300 µm. These three types of crystals were grown by mixing the protein solution and crystallization solution on an In Situ-1 Crystallization Plate (MiTeGen) using the NT8 drop setter (Formulatrix). The sitting-drop vapor-diffusion method was used for all crystallization experiments. All crystals were grown at 20°C.

**Figure 4:**
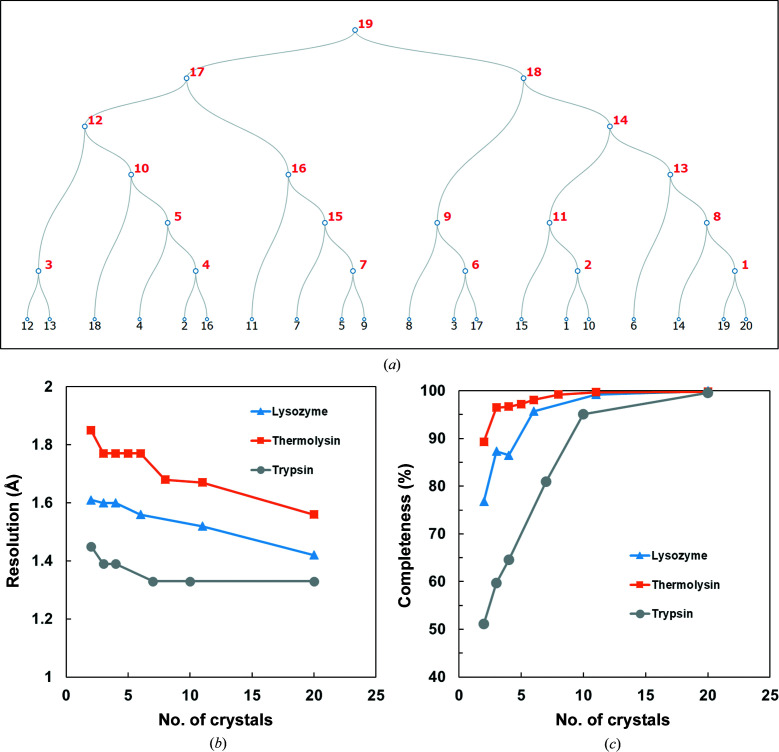
Results of small-wedge data collections. (*a*) Dendrogram for small-wedge measurement data of trypsin crystals. (*b*, *c*) Relationship between resolution (*b*) and completeness (*c*) and the number of measured crystals.

Diffraction data were measured using an EIGER X 4M (Dectris) at 20°C on BL26B1. The X-ray diffraction measurement conditions were as follows: a wavelength of 1.0000 Å, a beam size of 80 µm formed by a pinhole with a diameter of 80 µm, 20 crystals, 10° oscillation width, 0.1° per frame and 0.1 s per frame (a total of 10 s exposure for each crystal). The shortest camera length is 80 mm and the distance between the sample and the beam stop is 16 mm. Under these conditions, the highest and lowest measurable resolutions are 1.33 Å at the edge of the detector and 50 Å, respectively. The flux is 4.2 × 10^10^ photons s^−1^ at 1.0000 Å wavelength. In the case of data-set collection at 1.0000 Å wavelength, the maximum absorbed dose for each crystal calculated by *RADDOSE* version 2 corresponds to 49 kGy (Bury *et al.*, 2018 ▸). The data-collection statistics for these three samples are shown in Table 2 ▸. The resolution cutoffs of the processed data for lysozyme and thermolysin were determined based on the criterion of the CC_1/2_ value of the highest resolution bin being above ∼50%. The resolution cutoff of the processed data for trypsin was determined by the measurable highest resolution of the detector edge. The relationship between the resolution, completeness and number of crystals for each case measured by this system is also shown in Fig. 4 ▸. The clustering and merging of small-wedge data from multiple crystals were performed using *KAMO* (Yamashita *et al.*, 2018 ▸). Hierarchical clustering based on lattice parameters was performed using *BLEND* (Foadi *et al.*, 2013 ▸). Fig. 4 ▸(*a*) and Table 3 ▸ show the results of the data merging and the hierarchical clustering calculation from small-wedge data obtained from 20 trypsin crystals. In Table 3 ▸, the overall completeness and the overall multiplicity of each cluster were calculated at 1.33 Å resolution. In Figs. 4 ▸(*b*) and 4 ▸(*c*) we traced the clusters where the resolution or completeness increased efficiently and plotted them. The same calculations were performed for lysozyme and thermolysin and are shown in the same figures. Even with the increasing number of crystals (number of small-wedge data), we efficiently obtained high-resolution data (Fig. 4 ▸ *b*) and high completeness (Fig. 4 ▸ *c*). The space groups of the three samples all differed: lysozyme belongs to the tetragonal system, thermolysin to the hexagonal system and trypsin to the ortho­rhombic system.

## 4. *In situ* crystal soaking and structure analysis

The trypsin solution and crystallization solution were prepared as described above. Crystallization drops were prepared by mixing 250 nl trypsin solution and 250 nl crystallization solution on an In Situ-1 Crystallization Plate using an NT8 drop setter. One crystallization drop was made for each well of the plate. After one week, the compound solution was dispensed into the crystallized drops using an ECHO 650T acoustic liquid handler (Beckman Coulter; Sackmann *et al.*, 2016 ▸). The following compounds were used: benzamidine, 4-methoxybenzamidine, 4-bromobenzamidine, serotonin, 5-methoxytryptamine, 5-chlorotryptamine and tryptamine. In addition to the previous compounds, *N*-acetyl-5-hydroxy­tryptamine was used as a negative control. The compound solutions were dissolved in dimethyl sulfoxide (DMSO) and prepared at 500 m*M*, while 4-bromobenzamidine hydrochloride was prepared at 200 m*M*. 50 nl of each compound solution was then dispensed into 24 crystallization drops by the ECHO 650T. The compound concentrations in the drops were greater than 50 m*M* (greater than 20 m*M* for 4-bromobenzamidine), although the compound concentrations cannot be calculated accurately because the volume of the drop after vapor diffusion is dependent on the vapor pressures of the crystallization reagents. Diffraction measurements were performed after soaking at room temperature overnight (more than 12 h).

One crystal was selected from each crystallization drop (a total of 18–24 crystals were selected per compound). Diffraction data were collected from each crystal at a wavelength of 1.0000 Å using a beam formed by a pinhole with a diameter of 50 µm, covering 10° of oscillation width using 0.1° per frame and 0.1 s exposure per frame. The camera distance was set to 80 mm.

The data were processed by *KAMO*. Hierarchical clustering based on the correlation of reflection intensities was performed and the data were merged. Structural analyses were performed with the *DIMPLE* structure-analysis pipeline (Wojdyr *et al.*, 2013 ▸) using the molecular-replacement method with trypsin (PDB entry 1s0r) as the search model. Refinement and model modification were performed using *REFMAC* (Murshudov *et al.*, 2011 ▸) and *Coot* (Emsley *et al.*, 2010 ▸), respectively.

*In situ* diffraction measurements of trypsin crystals into which the compounds had been introduced by the acoustic liquid handler were carried out by collecting 10° data from each of 18–24 crystals, processing each wedge independently and merging the data. The results showed that the overall completeness of the data was 91.4–99.5% (Table 4 ▸). The crystal structures of the complexes of seven compounds other than *N*-acetyl-5-hydroxytryptamine were obtained from the eight compounds soaked into trypsin crystals. The resolution of the crystal structures of these complexes was 1.38–1.77 Å. The resolution cutoff was based on a CC_1/2_ value above ∼50% in the highest resolution shell.

**Figure 5:**
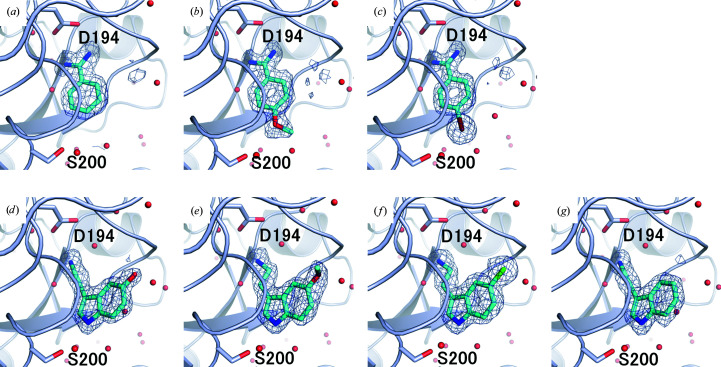
Crystal structures of the ligand-binding region of trypsin in complex with (*a*) benzamidine, (*b*) 4-methoxybenzamidine, (*c*) 4-bromobenzamidine, (*d*) serotonin, (*e*) 5-methoxytryptamine, (*f*) 5-chlorotryptamine and (*g*) tryptamine. The omit maps contoured at 3.0σ are shown as a mesh.

Benzamidine and its derivatives were bound to the S1 pocket of trypsin, and the amidyl group interacted electrostatically with Asp194 at the bottom of the S1 pocket (Fig. 5 ▸, Supplementary Fig. S1). In addition, tryptamine and its derivatives were bound to the S1 pocket via electrostatic interaction of the amino group with Asp194. The crystal structures of these complexes showed a high degree of similarity to the structures obtained from the data measured at room temperature, including the surrounding water molecules (Maeki *et al.*, 2020 ▸).

## 5. Discussion

*In situ* diffraction measurements are available as an application for laboratory-based X-ray sources (PX Scanner, Agilent Technologies; PlateMate, Rigaku; XtalCheck-S, Rigaku; Hargreaves, 2012 ▸). Several synchrotron-radiation facilities around the world are already equipped with crystallographic plate diffractometers. For example, the I04 beamline at Diamond Light Source (Douangamath *et al.*, 2013 ▸), X06DA at the Swiss Light Source (SLS; Bingel-Erlenmeyer *et al.*, 2011 ▸) and FIP-BM30A at the European Synchrotron Radiation Facility (ESRF; Jacquamet *et al.*, 2004 ▸; le Maire *et al.*, 2011 ▸) are equipped with a sample-exchange robot to hold a plate for measurement, while beamlines I03 and I24 at Diamond Light Source (Axford *et al.*, 2012 ▸), ID30B at ESRF (McCarthy *et al.*, 2018 ▸) and 19-ID at the Advanced Photon Source (APS; Michalska *et al.*, 2015 ▸) are equipped with a goniometer with a plate holder on the diffraction stage, and BL-17A at the Photon Factory (Yamada *et al.*, 2016 ▸) is equipped with a plate-holding stage on the diffraction stage. VMXi at Diamond Light Source is a beamline dedicated to *in situ* diffraction data collection using crystallization plates, and performs fully automated plate loading, mounting and diffraction measurements (Sanchez-Weatherby *et al.*, 2019 ▸). At VMXi, the mini-hutch and plate storage are located next to each other in the room, and the mini-hutch also has a temporary plate storage. The present *in situ* diffraction measurement system at SPring-8 is characterized by plate storage installed in restricted space conditions in the BL26B1 experimental hutch, and by the ability to mount crystallization plates automatically and perform diffraction measurements. As a unique feature of the plate storage, we adopted a commercially available plate rack; that is, the plate hotel of the Rock Imager system (Formulatrix). This is advantageous because the role of observation of crystal growth is performed by the system, and the plate hotels can easily be transported and stored for *in situ* diffraction data collection at the beamline. The plate storage and articulated robot can be relocated to the downstream position of the hutch when the plate diffraction experiment is not performed, so that the beamline can be used for multiple purposes such as humid air and glue-coating (HAG) experiments (Baba *et al.*, 2013 ▸, 2019 ▸).

In the data-set measurements, it is important to obtain data from a single crystal covering as wide an angle as possible to improve the completeness of the entire data set. On the other hand, using small-wedge data obtained from a large number of crystals and analyzing them as a single data set by merging the data has been recognized and utilized as a viable method (Baba *et al.*, 2021 ▸). The rotational angular range of our goniometer for a plate is narrower than that of diffractometers for crystallization plates in other facilities due to physical constraints. However, by increasing the number of data sets collected from crystals in different orientations to compensate for the restricted angular range, a sufficient data set for structural analysis can be obtained. In our experiment, we obtained 10° of data from fewer than 20 orthorhombic trypsin crystals and merged them to obtain data with near-full completeness (Fig. 4 ▸ *c*). In Fig. 4 ▸(*b*), the resolution of the merged data improves as the number of crystals for each protein increases in the *in situ* diffraction measurements using crystallization plates. This result has an analogy to the correlation between the number of crystals and the resolution in SS-ROX measurements (Hasegawa *et al.*, 2017 ▸, 2021 ▸). *In situ* diffraction measurements from multiple crystals using a microfluidic channel have also reportedly obtained a higher resolution and completeness of the data by increasing the number of crystals measured (Maeki *et al.*, 2020 ▸). If the crystals belong to a space group with high symmetry, the number of crystals to be measured can be further reduced. The *in situ* diffraction measurement system that we have developed needs multiple crystals for a data set, since we are using a small-wedge data-collection scheme. For this, using a crystallization plate that can supply many crystals without a crystal-fishing process is suitable. However, in our system this advantage was not fully utilized because the registration of the measurement queue of crystals was performed manually. Therefore, there is an urgent need to automate the various procedures leading to measurements. One possible way to improve efficiency would be to build a crystal visualization system before mounting the plates and register the crystal positions offline, which could lead to automated measurements.

To carry out compound-soaking experiments, it is necessary to evaluate the diffraction quality of the crystals under the measurement conditions in advance. Since compounds are often prepared as DMSO solutions, it is necessary to take into consideration the damage to crystals caused by DMSO during soaking. In a previous study, the damage caused to crystals by DMSO during soaking was investigated in detail in preliminary experiments (Collins *et al.*, 2017 ▸). In the present study, a 500 nl crystallization drop was mixed with 50 nl DMSO solution (final DMSO concentration of 10%) and soaked overnight at room temperature. 15 or more small-wedge data sets were then obtained from the ligand-free trypsin crystals soaked in DMSO, and greater than 90% completeness was obtained (Table 4 ▸). It was confirmed that more than 15 small-wedge data sets could be obtained from crystals soaked in 10% DMSO overnight with a resolution around 1.3 Å. In order to form ligand-bound trypsin crystals, the compounds in DMSO at a volume of 50 nl were introduced into a 500 nl crystallization drop and data were collected after overnight soaking. Similar preliminary experiments are necessary to obtain the crystal structure of the complex with the compound at a resolution better than ∼2.5 Å, which is sufficient to observe bound water molecules in order to discuss whether the interactions between the protein and the compound are based on hydrogen bonds or hydrophobic interactions.

In this study, we report the development of an *in situ* diffraction measurement system at SPring-8 that can collect small-wedge data from crystals in a crystallization drop on a crystallization plate at room temperature. The system eliminates the handling of crystals with cryo-loops and the infiltration of cryoprotectants. The structures of the compound complexes were obtained by collecting small-wedge diffraction data from crystals mixed with compound solutions dispensed by an acoustic liquid handler. Through future improvements to the automation and efficiency of the system such as crystal centering, it can be applied to research that requires efficiency, such as large-scale compound library screening at room temperature.

## Supplementary Material

PDB reference: trypsin, complex with benzamidine, 7wa0

PDB reference: complex with 4-methoxybenzamidine, 7wb2

PDB reference: complex with 4-bromobenzamidine, 7wb6

PDB reference: complex with serotonin, 7wb7

PDB reference: complex with 5-methoxytryptamine, 7wb8

PDB reference: complex with 5-chlorotryptamine, 7wb9

PDB reference: complex with tryptamine, 7wba

Supplementary Figure S1. DOI: 10.1107/S2053230X22005283/nw5113sup1.pdf

Supplementary Movie. Transporting and exchanging crystallization plates by the articulated robot. DOI: 10.1107/S2053230X22005283/nw5113sup2.mp4

## Figures and Tables

**Table 1:** Specifications of the goniometer for a plate

Main rotation axis	Horizontal pulse motor
Effective oscillation range (°)	−5 to 9
Rotation speed (deg s^−1^)	2.0
Rotation resolution (°)	0.005
Horizontal travel range (mm)	150
Vertical travel range (mm)	80
Travel range along the beam (mm)	20
Translation speed (mm s^−1^)	10

**Table 2:** Data-collection statistics of samples for equipment evaluation Values in parentheses are for the outer shell.

Name of protein	Lysozyme	Thermolysin	Trypsin
Beamline	BL26B1, SPring-8	BL26B1, SPring-8	BL26B1, SPring-8
Wavelength (Å)	1.0000	1.0000	1.0000
Space group	*P*4_3_2_1_2	*P*6_1_22	*P*2_1_2_1_2_1_
*a*, *b*, *c* (Å)	78.57, 78.57, 37.60	92.97, 92.97, 130.30	54.90, 58.36, 67.77
Resolution range (Å)	50.0–1.42 (1.51–1.42)	50.0–1.54 (1.63–1.54)	50.0–1.33 (1.38–1.33)
No. of crystals	20	20	20
Total no. of reflections	290097 (49786)	942733 (149647)	286972 (46735)
No. of unique reflections	22844 (3781)	49679 (7662)	50333 (7989)
Completeness (%)	100.0 (100.0)	99.7 (99.9)	99.6 (99.9)
Multiplicity	12.70 (13.17)	18.98 (19.53)	5.70 (5.85)
〈*I*/σ(*I*)〉	13.82 (1.01)	12.85 (1.07)	10.95 (1.83)
*R*_r.i.m._ † (%)	10.1 (231.5)	17.6 (313.4)	9.0 (92.6)
CC_1/2_	99.9 (51.0)	99.8 (49.7)	99.8 (71.6)

† *R*_r.i.m._ = *R*_meas_ = 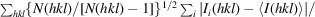 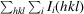 .

**Table 3:** Statistics of each cluster in the merged small-wedge data set of trypsin crystals

Cluster	No. of crystals	Cluster height	Completeness (overall) (%)	Multiplicity (overall)
19	20	11.4	99.8	7.4
17	10	6.4	93.9	3.9
18	10	6.8	96.8	3.8
14	7	2.2	89.3	2.9
12	6	1.7	82.5	2.7
16	4	2.9	68.2	2.2
10	4	0.9	75.9	2.0
13	4	1.8	76.5	1.9
11	3	1.1	60.5	1.8
15	3	2.3	61.2	1.8
8	3	0.7	65.1	1.7
9	3	0.9	67.7	1.6
5	3	0.7	69.6	1.6
7	2	0.7	47.9	1.5
2	2	0.4	48.4	1.5
4	2	0.6	51.5	1.4
3	2	0.5	51.2	1.4
1	2	0.2	52.3	1.4
6	2	0.7	52.5	1.4

**Table 4:** Data-collection and refinement statistics for trypsin crystals in complex with ligands Values in parentheses are for the outer shell.

Ligand	Benzamidine	4-Methoxybenzamidine	4-Bromobenzamidine	Serotonin	5-Methoxytryptamine	5-Chlorotryptamine	Tryptamine
Data-collection and merging statistics							
Date	29 June 2021	22 June 2021	29 June 2021	29 June 2021	22 June 2021	22 June 2021	22 June 2021
Beamline	BL26B1, SPring-8	BL26B1, SPring-8	BL26B1, SPring-8	BL26B1, SPring-8	BL26B1, SPring-8	BL26B1, SPring-8	BL26B1, SPring-8
Wavelength (Å)	1.0000	1.0000	1.0000	1.0000	1.0000	1.0000	1.0000
Space group	*P*2_1_2_1_2_1_	*P*2_1_2_1_2_1_	*P*2_1_2_1_2_1_	*P*2_1_2_1_2_1_	*P*2_1_2_1_2_1_	*P*2_1_2_1_2_1_	*P*2_1_2_1_2_1_
*a*, *b*, *c* (Å)	54.58, 58.13, 67.35	54.58, 58.30, 67.30	54.57, 58.28, 67.35	54.66, 58.17, 67.34	54.60, 58.38, 67.20	54.58, 58.22, 67.26	54.61, 58.14, 67.36
Resolution range (Å)	50.0–1.77 (1.88–1.77)	50.0–1.52 (1.61–1.52)	50.0–1.48 (1.57–1.48)	50.0–1.45 (1.54–1.45)	50.0–1.38 (1.46–1.38)	50.0–1.56 (1.65–1.56)	50.0–1.45 (1.54–1.45)
No. of crystals	16	9	13	13	15	16	10
Total No. of reflections	109342 (17523)	107283 (17236)	166794 (27140)	184387 (30773)	221492 (34007)	162352 (25015)	140789 (23301)
No. of unique reflections	21236 (3432)	30897 (4690)	36057 (5741)	38213 (6221)	44589 (6834)	30860 (4669)	37261 (6055)
Completeness (%)	98.3 (98.4)	91.4 (88.9)	98.7 (98.7)	98.5 (99.0)	99.3 (99.6)	98.8 (98.8)	96.1 (96.4)
Multiplicity	5.15 (5.11)	3.47 (3.68)	4.63 (4.73)	4.75 (4.95)	4.97 (4.98)	5.26 (5.36)	3.78 (3.81)
〈*I*/σ(*I*)〉	10.03 (2.36)	7.80 (2.48)	7.52 (2.06)	7.84 (1.80)	11.12 (1.60)	7.97 (1.79)	9.22 (2.14)
*R*_r.i.m._ (%)†	19.7 (149.4)	17.7 (121.2)	22.5 (126.8)	15.5 (120.7)	9.0 (129.4)	25.1 (207.5)	14.5 (133.1)
CC_1/2_	99.0 (49.9)	98.7 (53.5)	97.9 (51.6)	99.0 (53.5)	99.8 (52.8)	98.5 (49.5)	99.2 (50.1)
Overall *B* factor from Wilson plot (Å^2^)	16.7	11.2	16.7	14.0	15.4	15.0	13.7
Model refinement							
No. of reflections, working set	20176	29328	34218	36240	42442	29267	35353
No. of reflections, test set	1025	1532	1801	1925	2224	1550	1879
*R*_cryst_/*R*_free_ (%)	13.46/19.90	13.35/17.75	13.84/18.06	13.07/17.22	13.02/16.52	13.99/18.99	13.47/17.35
No. of non-H atoms							
Protein	1629	1629	1629	1629	1629	1629	1629
Ion	1	1	1	1	1	1	1
Ligand	13	15	14	17	18	17	16
Water	87	103	130	129	118	116	118
Total	1730	1748	1774	1776	1766	1763	1764
R.m.s. deviations							
Bond lengths (Å)	0.014	0.016	0.019	0.016	0.019	0.016	0.016
Bond angles (°)	1.772	1.732	1.949	1.746	1.867	1.798	1.817
Average *B* factor (Å^2^)	18.7	14.0	11.5	15.9	17.2	14.4	15.7
Ramachandran plot							
Favored region (%)	99.1	98.6	98.6	98.6	98.2	98.2	98.2
Additionally allowed (%)	0.9	1.4	1.4	1.4	1.8	1.8	1.8
Outliers (%)	0	0	0	0	0	0	0
PDB code	7wa0	7wa2	7wb6	7wb7	7wb8	7wb9	7wba

† *R*_r.i.m._ = *R*_meas_ = 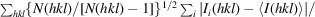 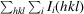 .
