# Supplementary material for: In situ crystal data-collection and ligand-screening system at SPring-8
Source: Acta Crystallogr F Struct Biol Commun. 2022 May 27;78(Pt 6):241–51. doi: 10.1107/S2053230X22005283 (PMC9158660; doi:10.1107/S2053230X22005283)
Supplement: Supplementary file 1 [file f-78-00241-sup1.pdf]

Acta Cryst F

STRUCTURAL BIOLOGY COMMUNICATIONS

Volume 78 (2022)

Supporting information for article:

In situ crystal data-collection and ligand-screening system at SPring-8

Hideo Okumura, Naoki Sakai, Hironori Murakami, Nobuhiro Mizuno, Yuki Nakamura, Go Ueno, Takuya Masunaga, Takashi Kawamura, Seiki Baba, Kazuya Hasegawa, Masaki Yamamoto and Takashi Kumasaka

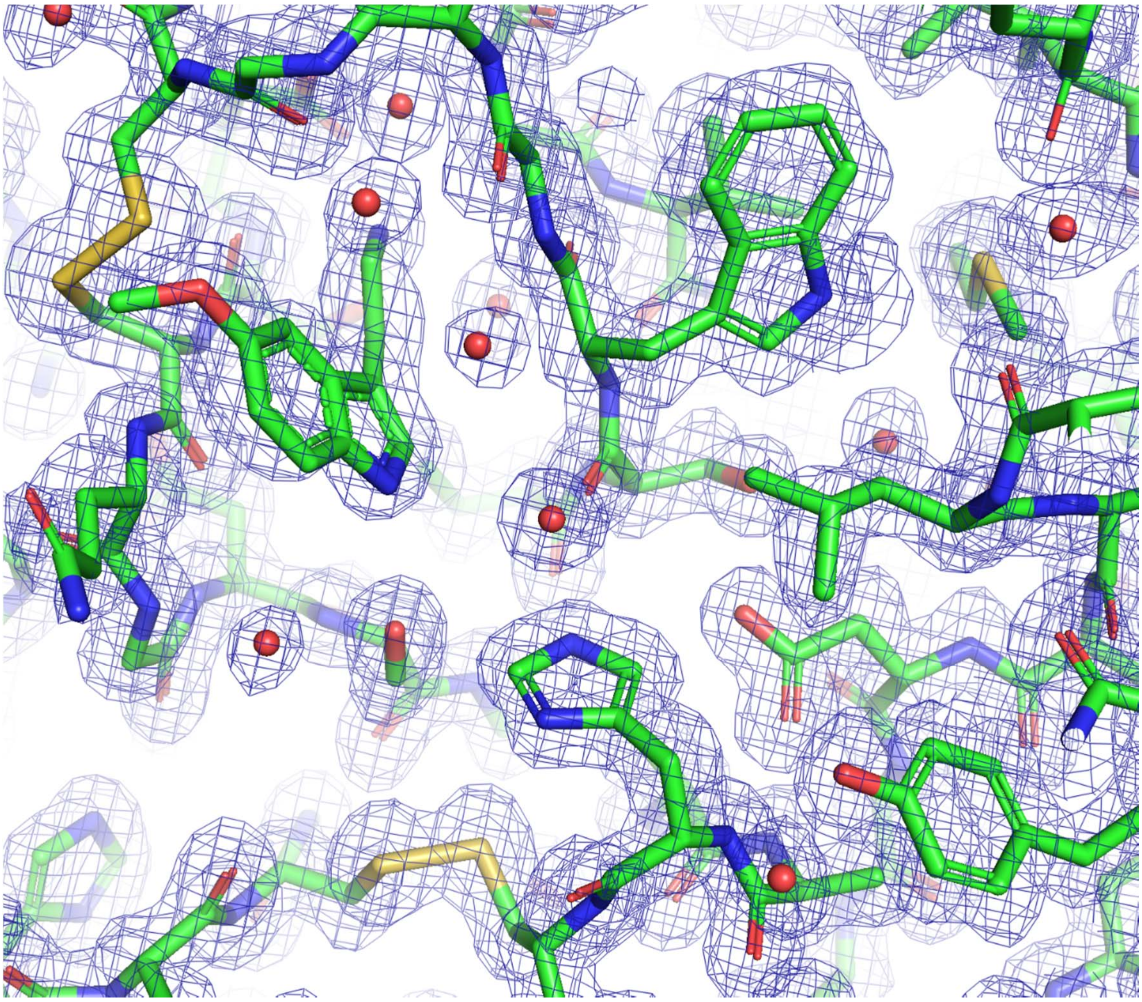

Figure S1 The 2Fo-Fc electron density map of trypsin in complex with 5-Methoxytryptamine contoured at 1.0 σ.
